# Deep Learning Analysis of the Adipose Tissue and the Prediction of Prognosis in Colorectal Cancer

**DOI:** 10.3389/fnut.2022.869263

**Published:** 2022-05-11

**Authors:** Anqi Lin, Chang Qi, Mujiao Li, Rui Guan, Evgeny N. Imyanitov, Natalia V. Mitiushkina, Quan Cheng, Zaoqu Liu, Xiaojun Wang, Qingwen Lyu, Jian Zhang, Peng Luo

**Affiliations:** ^1^Department of Oncology, Zhujiang Hospital, Southern Medical University, Guangzhou, China; ^2^College of Biomedical Engineering, Southern Medical University, Guangzhou, China; ^3^Department of Information, Zhujiang Hospital, Southern Medical University, Guangzhou, China; ^4^Department of Tumor Growth Biology, N.N. Petrov Institute of Oncology, St. Petersburg, Russia; ^5^Department of Neurosurgery, Xiangya Hospital, Central South University, Changsha, China; ^6^Department of Interventional Radiology, The First Affiliated Hospital of Zhengzhou University, Zhengzhou, China; ^7^First People's Hospital of Chenzhou City, Chenzhou, China

**Keywords:** deep learning, adipose tissue, colorectal cancer, prognosis, hematoxylin and eosin

## Abstract

Research has shown that the lipid microenvironment surrounding colorectal cancer (CRC) is closely associated with the occurrence, development, and metastasis of CRC. According to pathological images from the National Center for Tumor diseases (NCT), the University Medical Center Mannheim (UMM) database and the ImageNet data set, a model called VGG19 was pre-trained. A deep convolutional neural network (CNN), VGG19CRC, was trained by the migration learning method. According to the VGG19CRC model, adipose tissue scores were calculated for TCGA-CRC hematoxylin and eosin (H&E) images and images from patients at Zhujiang Hospital of Southern Medical University and First People's Hospital of Chenzhou. Kaplan-Meier (KM) analysis was used to compare the overall survival (OS) of patients. The XCell and MCP-Counter algorithms were used to evaluate the immune cell scores of the patients. Gene set enrichment analysis (GSEA) and single-sample GSEA (ssGSEA) were used to analyze upregulated and downregulated pathways. In TCGA-CRC, patients with high-adipocytes (high-ADI) CRC had significantly shorter OS times than those with low-ADI CRC. In a validation queue from Zhujiang Hospital of Southern Medical University (Local-CRC1), patients with high-ADI had worse OS than CRC patients with low-ADI. In another validation queue from First People's Hospital of Chenzhou (Local-CRC2), patients with low-ADI CRC had significantly longer OS than patients with high-ADI CRC. We developed a deep convolution network to segment various tissues from pathological H&E images of CRC and automatically quantify ADI. This allowed us to further analyze and predict the survival of CRC patients according to information from their segmented pathological tissue images, such as tissue components and the tumor microenvironment.

## Introduction

Colorectal cancer (CRC) is a common global disease that is the third most common type of cancer and the second leading cause of cancer deaths worldwide (1–4). In 2018, there were 1.8 million new cases of CRC (1). Thus, CRC seriously affects and threatens global health and quality of life.

The prognosis of CRC patients depends on the tumor node metastasis (TNM) stage of the cancer and, for patients with early-stage CRC, whether they receive curative surgical interventions. CRC prognosis is also influenced by epigenetics and the TIME, both of which cause CRC heterogeneity (5). Despite the poor prognosis and high recurrence rate of CRC, potential biomarkers have helped to improve the prognosis of CRCs (6–10). It has been shown that the lipid microenvironment surrounding the tumor is associated with the occurrence, development, and metastasis of CRC (11–13). In addition, obesity is associated with increased incidence and mortality of CRC (14–16). Adipocytes (ADI) in the TIME can act directly as energy providers and metabolic regulators to promote the proliferation, invasion, and even drug resistance of CRC (14). However, the lipid microenvironment of CRC is complex, and there is considerable heterogeneity among individuals; therefore, a suitable method is needed to evaluate the lipid microenvironment of CRC patients to more accurately predict their prognoses.

In recent years, the rise of the CNN in the artificial intelligence field has allowed for extraction of pathological image features by automatic model extraction as opposed to traditional manual design (17–19). Furthermore, the CNN has promoted quantitative analysis of pathological images, as opposed to only qualitative analysis. When quantitative image data is combined with accurate clinical data and follow-up information, a systematic model of prognosis and survival can be constructed, and the postoperative status of patients can be predicted to develop a personalized treatment plan (20, 21). Kather et al. (22) used a CNN to study the auxiliary diagnoses of CRC pathological sections and extract texture features from a large number of H&E sections. The features were then input into a classifier to predict the tissue type. Using this approach, recognition accuracy could exceed 94%. Xu et al. (23) trained a deep learning CNN model on ImageNet, and it was superior to manual classification and segmentation of colon cancer (CRC) histopathological images. Deep learning can further predict the survival status of patients by counting the number of mitotic cells in the CRC image, quantifying immune cell infiltration, evaluating the degree of tumor differentiation, and characterizing the surrounding tissues (24–32).

In the present study, we segmented various tissues from H&E-stained pathological images of CRC by constructing a deep convolution network. We used this network to further analyze and predict the survival status of CRC patients according to their segmented pathological tissue image information, including TIME composition. We recommend that quantified image features are useful for predicting CRC patient prognosis and informing treatment plans.

## Methods

### Construction of the Neural Network Model

According to published literature, we used a data set containing more than 100,000 H&E image patches and corresponding categories, which we obtained by non-overlapping slicing of pathological images in the NCT and UMM databases. Patches of histological images of CRC were divided into nine categories: adipose tissue (ADI), background (BACK), debris (DEB), hematoxylin-eosin (HE), lymphocytes (LYM), mucus (MUC); smooth muscle (MUS), National Center for Tumor Diseases (NCT), normal colon mucosa (NORM), cancer-associated stroma (STR), and colorectal adenocarcinoma epithelium (TUM) (22, 33). Based on this data set and the VGG19 model that was pre-trained on the ImageNet data set (www.image-net.org), we trained a deep CNN by migration learning. We preliminarily constructed a classifier called VGG19_CRC_ based on histological images of CRC, and we used this classifier to classify tumor and non-tumor sections of colorectal tissue. The classifier obtained an accuracy of 97.3% on the classification of the verification set; therefore, use of this classifier allowed for rapid organization of CRC images into the aforementioned categories.

### TCGA-CRC Queue

The 862 H&E images of COAD and READ patients (Stages: I-IV) were downloaded from the Genomic Data Commons (GDC) database. To study the CRC queue, we combined the TCGA-COAD queue and the TCGA-READ queue into the TCGA-CRC queue. Because the size of full slice images in the TCGA-CRC queue is very large, the full slice images cannot be directly used as inputs for the neural network (22). Therefore, we cut the full slice images into image blocks with a resolution of 224 pixels x 224 pixels for verification and testing. To maintain similar feature distributions for the 100,000 pathological image blocks used for training VGG19_CRC_, we chose 0.5 as the microns per pixel (mpp) for generating pathological image blocks, which allowed each pathological section to generate tens to thousands of pathological blocks. The cuts were then carried out as follows: 1) Cuts with too large of a background proportion were removed. For this step, each image block sized 224 pixels x 224 pixels was pooled into a 1 x 1 image block by global average, and the gray matrix of the 1 x 1 pixel after pooling was determined. If the red green blue (RGB) value of the pixel gray matrix was >200, the corresponding 224 pixel x 224 pixel was considered background and eliminated from the block generated from the pathological section. 2) Color normalization: Here, we used the “normalize” method in the torchvision framework to pool the gray matrix of the input cut data into a distribution similar to that of the training data. This allowed us to identify similar feature distributions and to improve VGG19CRC's discrimination accuracy for tumor and non-tumor tissues. The preprocessed slices were then input into the VGG19CRC model, and the category of each slice was the output. Finally, we calculated the proportions of the nine tissue categories in each patient's H&E image. Detailed clinical information for TCGA-CRC is provided in Supplementary Table 1.

### External Validation Set Queues and ADI Calculations

H&E images of 660 CRC patients were collected from Zhujiang Hospital of Southern Medical University; these patients were referred to as “Local-CRC1”. Detailed clinical information for Local-CRC1 is provided in Supplementary Table 2. We also collected H&E images of 164 CRC patients from First People's Hospital of Chenzhou; these patients were referred to as “Local-CRC2”. Detailed clinical information for Local-CRC2 is provided in Supplementary Table 3. For the local queues, we adopted the same image preprocessing method as we did for the TGCA-CRC queue. The H&E images were applied to the trained VGG19_CRC_ model, and we obtained the proportions of the nine tissue categories in each patient's H&E image. Details of patient recruitment for the two independent cohorts are provided in the “Supplementary Methods.”

### Evaluation of ADI Prognostic Value

In the TCGA-CRC, TCGA-COAD, TCGA-READ, TCGA-CRC-Male, and TCGA-CRC-Female queues, the “surv_cutpoint” function in the R package “survminer” was used to divide the patients in each queue into high-ADI and low-ADI groups based on their median ADI values. We then used the Kaplan-Meier (KM) analysis method to compare the OS of patients in the high-ADI group and the low-ADI group.

### Immune Infiltration Analysis

In the TCGA-CRC cohort, we used the xCell (34) and MCP-Counter (35) algorithms to evaluate the patients' immune cell scores based on the expression data. We obtained the immune scores of the TCGA-CRC patients from published literature (36).

### Statistical Analysis

We used RNA-seq data for the TCGA-CRC queue downloaded from the Genomic Data Commons (GDC) database (https://portal.gdc.cancer.gov/) and the GO-BP, GO-CC, GO-MF, KEGG, and REACTOME gene sets downloaded from the Molecular Signatures Database (MsigDB) (37). We then used the R package “clusterprofiler” to perform the GSEA on the TCGA-CRC gene expression data (38). We expressed the upregulation or downregulation of each pathway by the enrichment fraction, and statistical differences were communicated using *P*-values. *P*-values < 0.05 indicate significant upregulation or downregulation. For the KM analyses, the log-rank *P*-value was used to express statistical differences. We also used the R package “ComplexHeatmap” (39) to visualize the heat map and the R packages “survival” (40) and “survminer” (41) for the survival analysis. For the ssGSEA (42), which is an extension of GSEA and standardizes the ranks of gene expression values for given samples, the empirical cumulative distribution function was used to calculate enrichment scores (ESs). We used the R package “GSVA” to perform the ssGSEA on the expression profile data of the TCGA-CRC patients (42). In the present study, all analyses were conducted in Python (Version 3.6) or R software (Version 3.6.3).

## Results

### Non-tumor Multi-Tissue Type Decomposition of CRC Pathological Images Based on a Deep Convolution Neural Network

**Figure 1:**
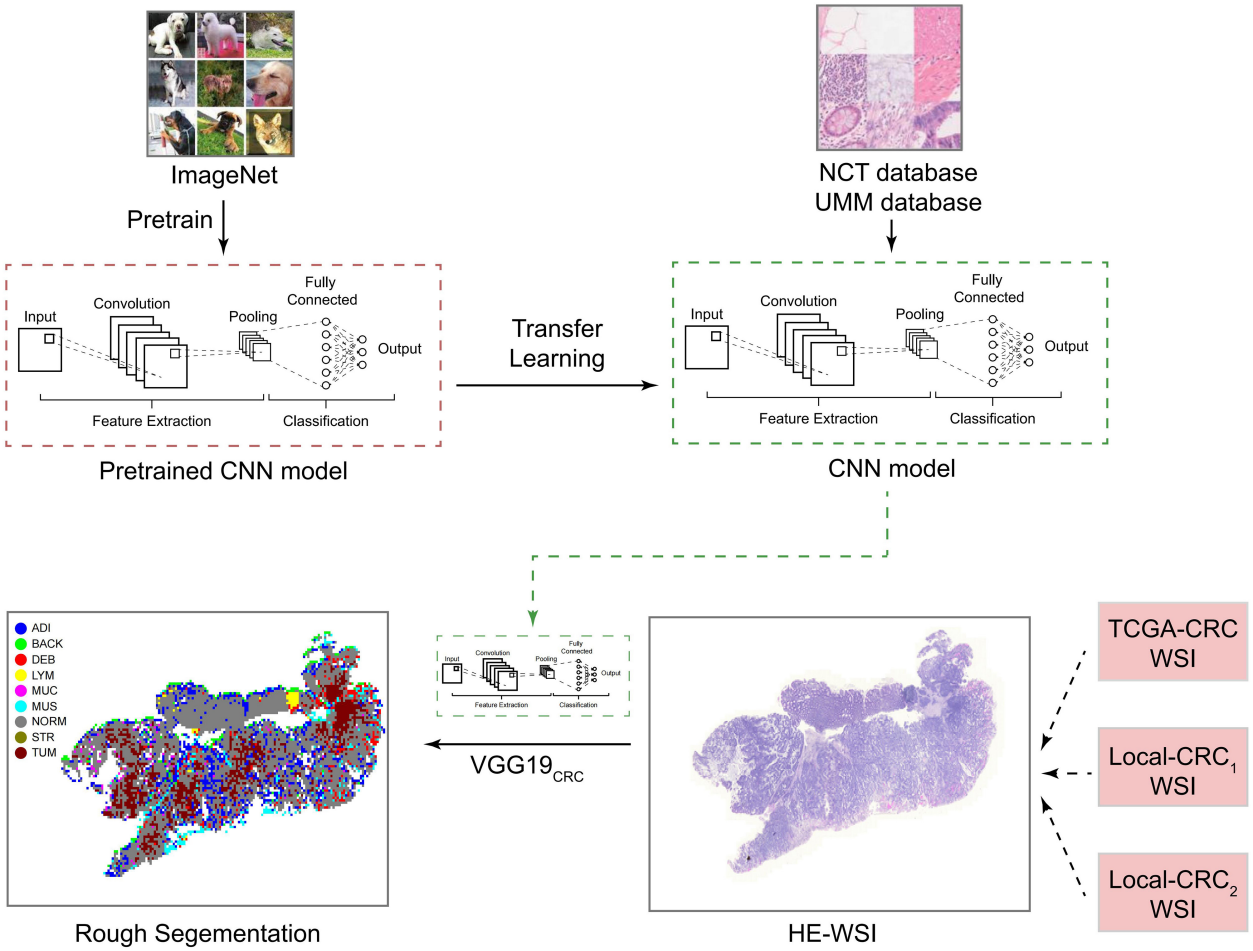
Study design for the development and application of the CNN model. CNN, convolutional neural network; H&E, hematoxylin and eosin; WSI, whole-slide image; ADI, adipose; BAC, background; DEB, debris; LYM, lymphocyte aggregates; MUC, mucus; MUS, muscle; NOR, normal mucosa; STR, stroma; and TUM, tumor epithelium.

Based on the methods of Kather et al. (22), we extracted 100,000 H&E images (http://dx.doi.org/10.5281/zenodo.1214456) and corresponding data sets from 86 CRC tissue slices in the NCT and UMM databases. We then pre-trained the VGG_19_ algorithm on the ImageNet data set (www.image-net.org) and Kather data set. From this, we obtained the classification levels of nine organization categories. We then trained a CNN through transfer learning to classify different tissue types in pathological CRC images (VGG19CRC). We next used the preliminarily constructed VGG19_CRC_ to segment whole-slide images (WSIs) from the TCGA-CRC, Local-CRC1, and Local-CRC2 queues into several 224 x 224 images, and these image blocks were input into the VGG19_CRC_ model to generate prediction probability. The final classification of each image block was set as the organization category with the maximum probability. Further details on this process are provided in Figure 1.

**Figure 2:**
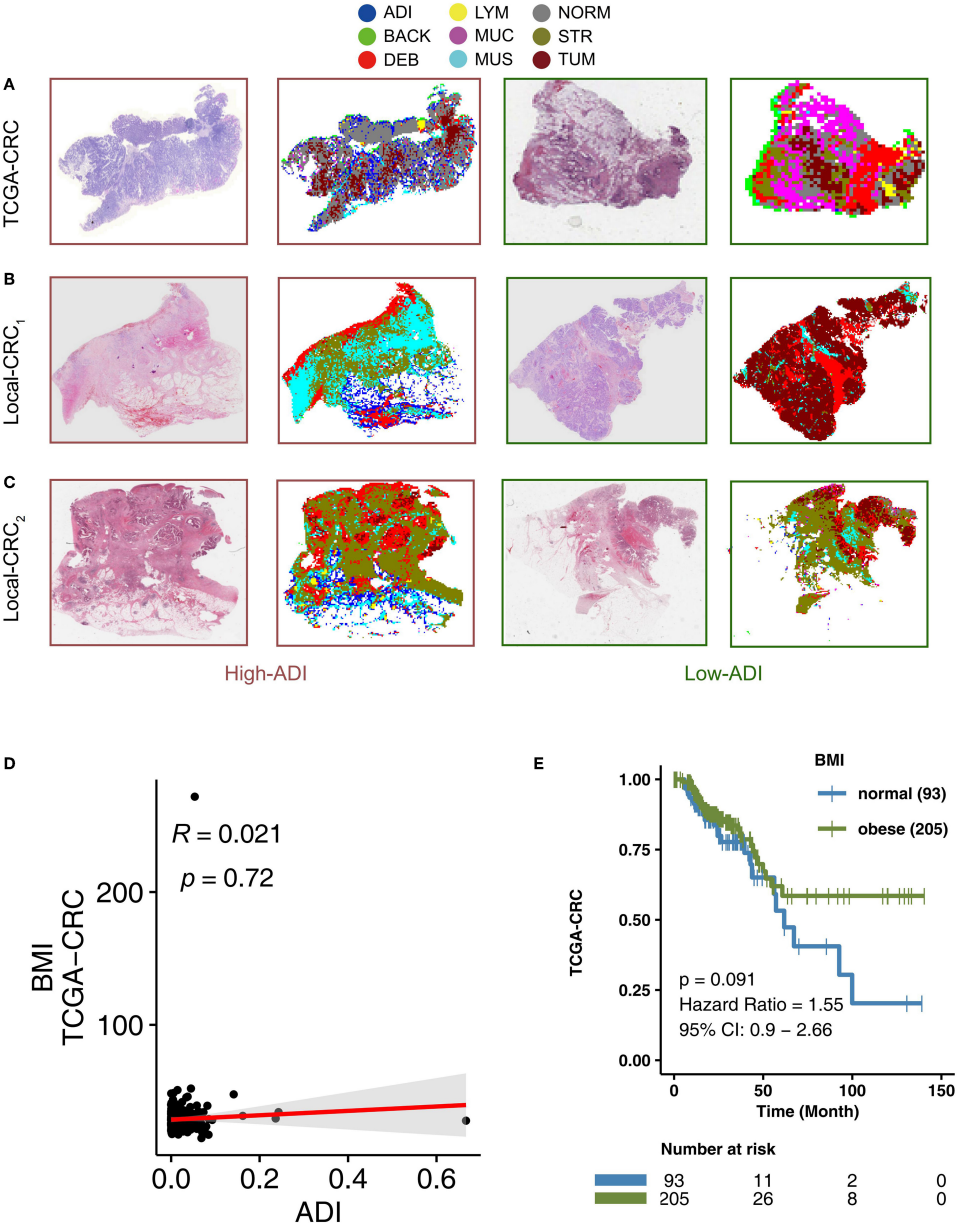
The association between BMI and clinical prognosis of TCGA-CRC patients. Examples of high-ADI and low-ADI H&E-stained WSIs and corresponding segmented results from the TCGA-CRC cohort **(A)**, Local-CRC1 **(B)**, and Local-CRC2 **(C)**. **(D)** The correlation between BMI and ADI in the TCGA-CRC cohort (Spearman correlation). **(E)** Overall survival for subjects grouped according to BMI subclass (obese and normal). H&E, hematoxylin–eosin; WSI, whole-slide image; and ADI, adipose.

To determine adipose scores for H&E images using the VGG19_CRC_ model, the following process was applied. H&E stained images in the TCGA-CRC, Local-CRC1, and Local-CRC2 queues were cut into several fixed-size slices, and these slices were applied to the trained VGG19 model to classify the slice type. The ADI score was then determined based on the proportion of slices that were characterized as ADI by the trained VGG19_CRC_ model. For example, if a single patient had 1,000 slices, and 200 slices were classified as ADI by the VGG19_CRC_ model, this patient's ADI would be 0.2. In addition, the VGG19_CRC_.model could segment H&E images well. Representative examples of segmentation for when the VGG19_CRC_ model was applied to high-ADI and low-ADI samples are shown in Figures 2A–C (Figure 2A: TCGA-CRC; Figure 2B: Local-CRC1; Figure 2C: Local-CRC2).

### BMI Is Not Associated With CRC Patient Prognosis

Adipose tissue is an important component of obesity. To explore the relationship between adipose tissue scores in H&E images and corresponding Body Mass Indices (BMI), we performed a correlation analysis. We found that there was no statistically significant correlation between ADI and BMI in TCGA-CRC (*R* = 0.021; *P* = 0.72) (Figure 2D). Some studies have suggested that obesity is a risk factor for CRC recurrence and death (43–45). In the TGCA-CRC cohort, we divided the patients into groups according to the WHO definition of obesity (46) (obesity: BMI > 25; normal: BMI ≤25). We then used KM analysis to compare the differences in OS between obese patients and normal patients. We found that there was no significant difference in OS between obese patients and normal patients in the TCGA-CRC cohort (Figure 2E).

### Higher ADI Is Associated With Worse OS in CRC Patients

**Figure 3:**
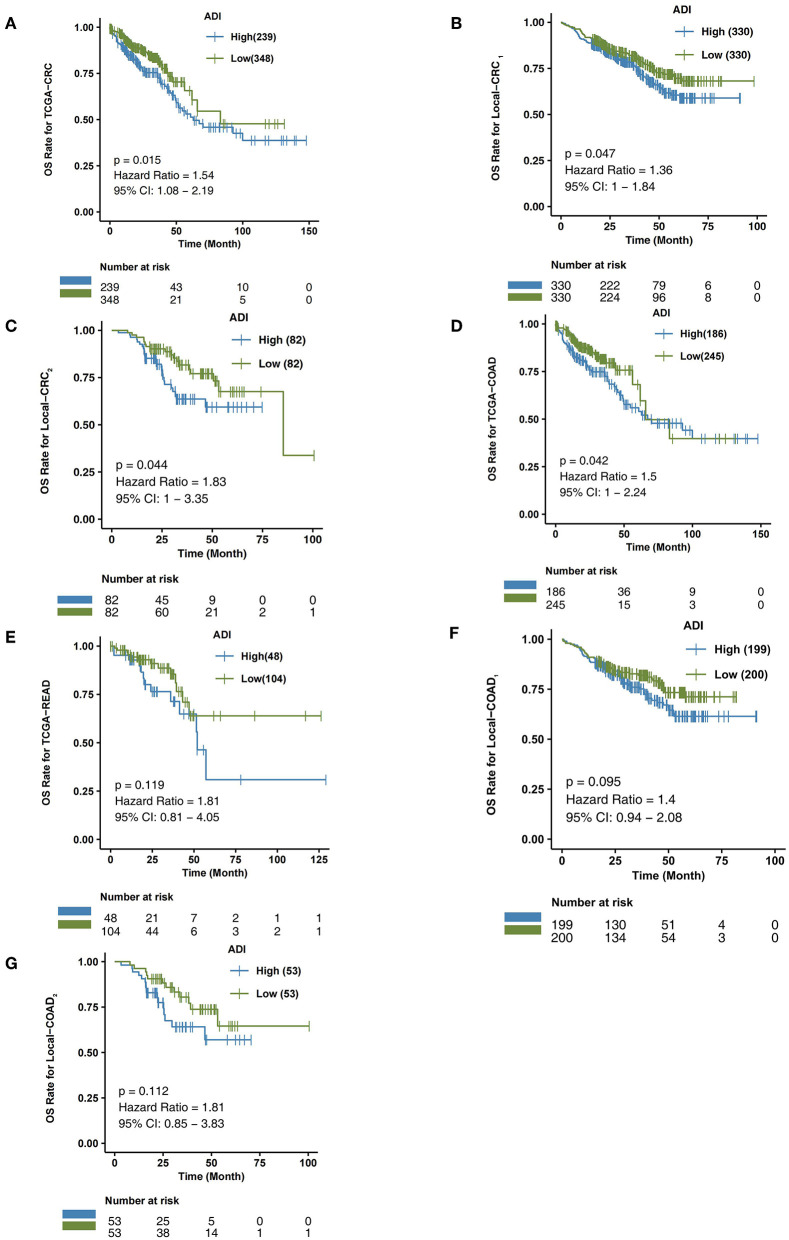
The association between ADI and OS of CRC patients. Overall survival for subjects grouped according to ADI subclass (high-ADI and low-ADI) in the TCGA-CRC cohort **(A)**, Local-CRC1 **(B)**, Local-CRC2 **(C)**, TCGA-COAD **(D)**, TCGA-READ **(E)**, Local-COAD1 **(F)**, and Local-COAD2 **(G)**.

In the TCGA-CRC queue, patients with high-ADI CRC had significantly shorter OS times (log-rank *P* = 0.015; HR = 1.54; 95%Cl: 1.08–2.19; Figure 3A), compared to those with low-ADI CRC. To further verify the predictive effect of ADI on CRC prognosis, we also retrospectively collected H&E images from the Local-CRC1 and Local-CRC2 verification sets. In the Local-CRC1 cohort, CRC patients with high-ADI (n = 330) had worse OS than CRC patients with low-ADI (log-rank *p* = 0.047; HR = 1.36; 95%Cl: 1–1.84; Figure 3B). In the Local-CRC2 queue, patients with low-ADI CRC (*n* = 82) had significantly longer OS time (log-rank *P* = 0.044; HR = 1.83; 95%Cl: 1–3.35; Figure 3C) than those with high-ADI CRC. These results suggest that ADI is a potential biomarker for predicting the prognosis of CRC patients.

### ADI Is Associated With the Prognosis of CRC Subgroup Patients

We then explored the predictive effect of ADI on the prognosis of CRC subgroup patients, including males, females, and the COAD and READ CRC subtypes.

In the TCGA-COAD queue, patients with high-ADI COAD had significantly lower OS time than those with low-ADI COAD (log-rank *P* = 0.042; HR = 1.5; 95%Cl: 1–2.24; Figure 3D). In the TCGA-READ queue, however, we found no differences in OS time between patients with high-ADI READ and patients with low-ADI READ (log-rank *P* = 0.119; HR = 1.81; 95%Cl: 0.81–4.05; Figure 3E).

**Figure 4:**
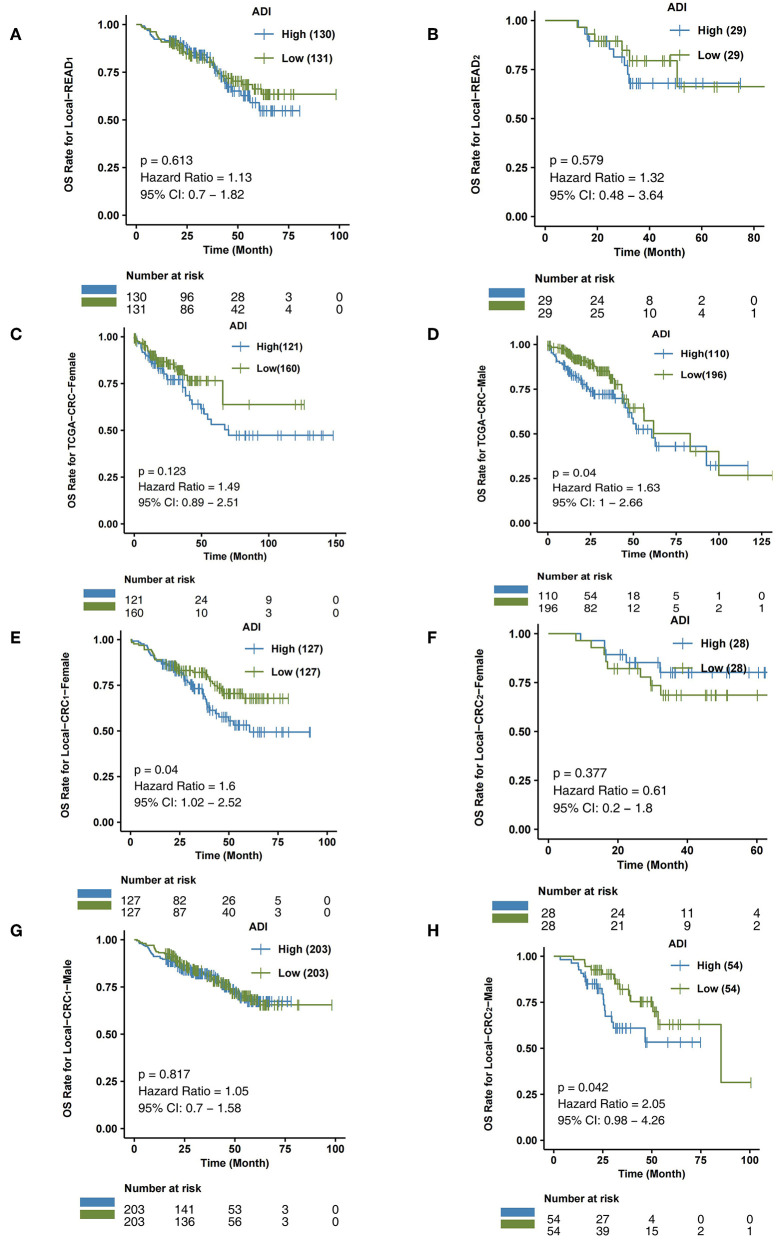
The association between ADI and OS of CRC subgroups. Overall survival for subjects grouped according to ADI subclass (high-ADI and low-ADI) in the queues Local-READ1 **(A)** and Local-READ2 **(B)**, TCGA-CRC-Female **(C)**, TCGA-CRC-Male **(D)**, Local-CRC1-Female **(E)**, Local-CRC2-Male **(F)**, Local-CRC1-Female **(G)**, and Local-CRC2-Male **(H)**.

To further verify the predictive effect of ADI on the prognosis of CRC subtype patients, we analyzed CRC patients from the independent verification sets Local-CRC1 and Local-CRC2. According to histological type and gender, we divided the two independent verification sets into Local-COAD1, Local-COAD2, Local-READ1, Local-READ2, Local-CRC1-Female, and Local-CRC1-Male. In Local-COAD1, we found that patients with high-ADI tended to have shorter OS times than patients with low-ADI, although this difference was not statistically significant (log-rank *P* = 0.095; Figure 3F). Similarly, in Local-COAD2, compared with low-ADI patients, high-ADI patients tended to have shorter OS times (log-rank *P* = 0.112; Figure 3G). In local-READ1 and local-READ2, we found no significant difference in OS time between the high-ADI patients and the low-ADI patients (Figure 4A: log-rank *P* = 0.613; Figure 4B: log-rank *P* = 0.579).

Within the TCGA-CRC cohort, we analyzed the relationship between ADI and prognosis for male and female patients. As shown in Figure 4C, ADI had no predictive effect on OS time in TCGA-CRC female patients (log-rank *P* = 0.123). The male patients with high-ADI had significantly shorter OS times than those with low-ADI (log-rank *P* = 0.04; HR = 1.63; 95%Cl: 1–2.66; Figure 4D). In the Local-CRC1-Female subgroup, we found that patients with high-ADI had significantly lower OS times than patients with low-ADI (Figure 4E: log-rank *P* = 0.04; HR = 1.6; 95%Cl: 1.02–2.52). However, within the Local-CRC2-Female subgroup, there was no statistically significant difference in OS time between the high-ADI and low-ADI patients (Figure 4F; log-rank *P* = 0.377). In the Local-CRC1-Male subgroup, there was no statistically significant difference in the OS times of high-ADI and low-ADI patients (Figure 4G; log-rank *P* = 0.817). In the Local-CRC2-Male subgroup, the clinical prognosis of patients with high-ADI was significantly worse than that of patients with low-ADI (Figure 4H).

### Immune Infiltration Differences Between High-ADI and Low-ADI CRC Patients

**Figure 5:**
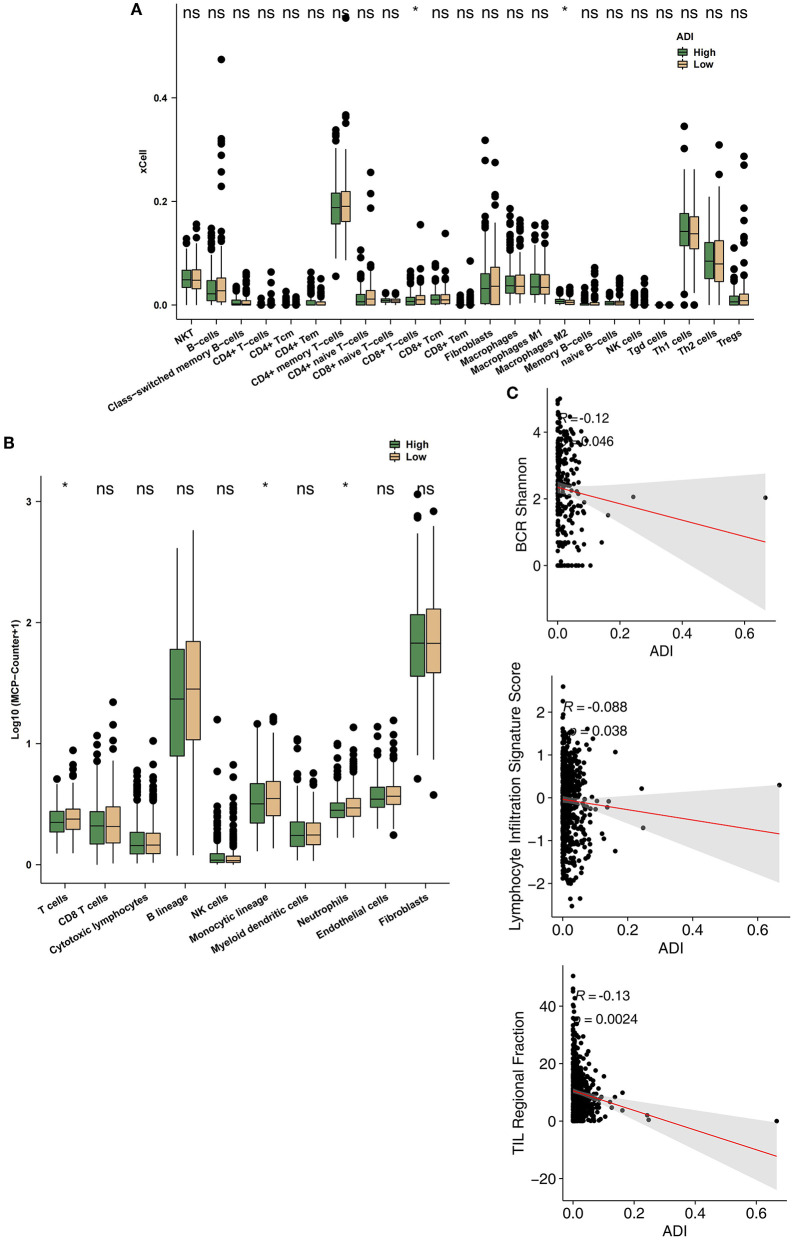
Comparison of immune infiltration between the high-ADI and low-ADI groups. **(A)** Comparison of immune cell infiltration estimated by xCell between the high-ADI and low-ADI groups. **(B)** Comparison of immune cell infiltration estimated by MCP-Counter between the high-ADI and low-ADI groups. **(C)** Comparison of immune-related signature scores between the high-ADI and low-ADI groups. (*P < 0.05 Mann-Whitney U test). ns: not significant.

To further explore differences in the TIMEs of high-ADI and low-ADI CRC patients, we used the xCell and MCP-Counter algorithms to evaluate gene expression in the TIME. As shown in Figure 5A, CD8+ T cells were significantly enriched in the immune microenvironments of low-ADI patients compared with those of high-ADI patients. M2 macrophages were significantly enriched in the TIMEs of high-ADI patients compared to those of low-ADI patients. Results from MCP-Counter (Figure 5B) showed that the high-ADI group had significantly fewer activated lymphocytes, such as T cells, neutrophils, and monocytes, than the low-ADI group. We then further explored the relationship between ADI and immune infiltration scores. We found significant negative correlations between ADI and BCR Shannon, lymphocyte infiltration signature score, and TIL regional fraction (Figure 5C; all *P* < 0.05; all R > 0).

### Differences in Signaling Between High-ADI and Low-ADI CRC Patients

**Figure 6:**
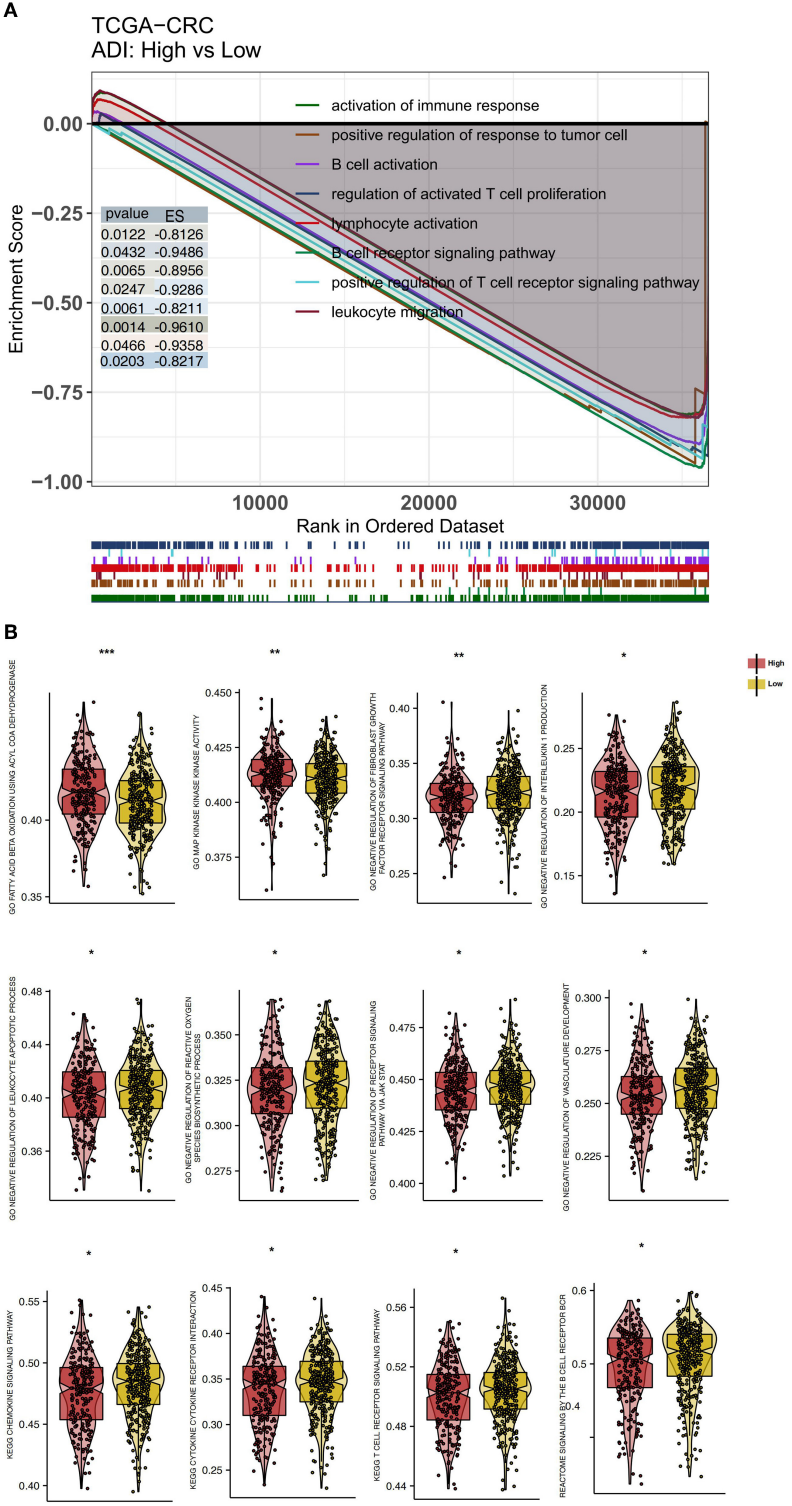
Differentially enriched biological functions of the high-ADI and low-ADI groups of the TCGA-CRC cohort, identified by transcriptome analysis. **(A)** The results of the GSEA. The color of the curve corresponds to the font color of each pathway. GSEA of hallmark gene sets were downloaded from the Molecular Signatures Database (MSigDB). Each run was performed with 1,000 permutations. Enrichment results with significant differences between the high-ADI and low-ADI tumors are shown. **(B)** Boxplot depicting the significant mean differences in ssGSEA scores between the high-ADI and low-ADI tumors in the TCGA-CRC queue. ^*^P < 0.05; ^*^^*^P < 0.01; ^*^^*^^*^P < 0.001.

To explore differences in the activity of pathological signaling pathways between the high-ADI low-ADI groups, we used the GSEA algorithm to calculate and compare enrichment scores (ESs) of pathological pathways. In both the training set and verification set of TCGA-CRC, we found that the following pathways were significantly downregulated in the high-ADI group compared to the low-ADI group: immune activation; lymphocyte activation; leukocyte migration; B cell activation; regulation of activated T cell proliferation; positive regulation of T cell receptor signaling; positive regulation of anti-tumor immunity; and B cell receptor signaling (*P* < 0.05, ES < 0; Figure 6A; Supplementary Table 1). We also used the ssGSEA algorithm to calculate the activity of signaling pathways of each patient in the TCGA-CRC queue. For this, we analyzed differences between the high-ADI and low-ADI groups in the TCGA-CRC queue using the “limma test” method. We found that the following pathways were significantly downregulated in the high-ADI group compared to the low-ADI group: cytokine production, chemokine signaling, TCR and BCR signaling, negative regulation of lymphocyte apoptosis, negative regulation of fibroblast growth factor receptor (FGFR) signaling, negative regulation of angiogenesis, negative regulation of reactive oxygen species (ROS) synthesis, and negative regulation of JAK/STAT signaling (Figure 6B). On the contrary, the MAPK signaling pathway was significantly upregulated in the high-ADI group due to fatty acid oxidation (Figure 6B).

## Discussion

Understanding and characterizing CRC is very important for evaluating the prognosis of patients and informing treatment decisions (47). Previous studies have shown that clinical variables, histopathological parameters, and molecular characteristics are associated with the clinical prognosis of patients (48, 49). For example, Wulczyn et al. constructed a deep learning system (DLS) score that can predict the disease-specific survival (DSS) of stage II and stage III CRC patients by analyzing H&E images. They found that CRC patients with higher DLS scores had significantly shortened DSS times (47). In addition, Zhao et al. (50) trained a CNN model using transfer learning and quantified the tumor-stroma ratio (TSR) after segmenting H&E images of CRC patients. They found that the OS time of the high-TSR group was significantly lower than that of the low-TSR group (*P* < 0.001, HR = 1.79). In the present study, we used a CNN model to segment CRC images from the TCGA-CRC queue and two Local-CRC queues and automatically quantified the adipose tissue scores of the H&E images. Further analysis showed that the ADI score constructed by the CNN could predict the OS of CRC patients. CRC patients in the high-ADI group had significantly lower OS times than patients in the low-ADI group. In the subgroup analysis, ADI could also be used as a prognostic marker for COAD, READ, CRC-Male, and CRC-Female patients. Across these groups, patients with lower ADI had significantly improved OS times. To our knowledge, this is the first study to establish a deep learning model based on WSI for automatic quantification of ADI. We have verified the predictive effectiveness of ADI on prognosis in several independent queues. Our method can quantify ADI from H&E-stained histological images efficiently and accurately, thus eliminating deviation caused by traditional visual evaluation and reducing the workload for pathologists.

**Figure 7:**
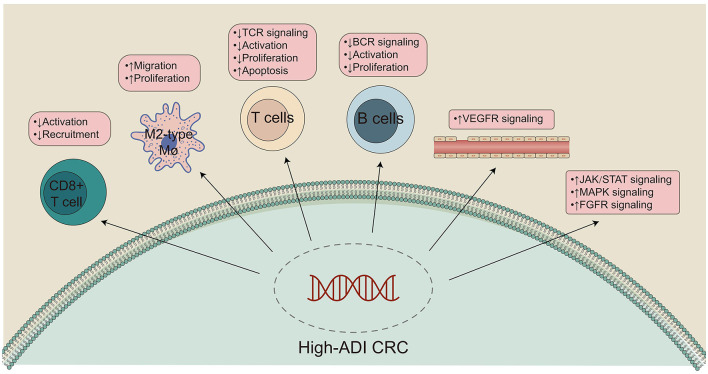
Potential mechanism underlying the prognostic value of high-ADI.

The role of adipose tissue in the occurrence, development, invasion, and metastasis of CRC is receiving increased research attention (14). In addition to constructing a biomarker, ADI, that can predict CRC patient prognosis, we hope to clarify the molecular mechanisms by which high-ADI negatively impacts patient prognosis (Figure 7). GSEA and ssGSEA were used to compare the upregulation or downregulation of pathological pathways between high-ADI and low-ADI CRC patients. We found that anti-tumor immunity pathways, including cytokine production, chemokine signaling, TCR signaling, BCR signaling, and negative regulation of lymphocyte apoptosis, were significantly downregulated in the high-ADI group (51, 52). Kawamura et al. (53) have shown that cytotoxic T lymphocyte (CTL) responses to peptide vaccines can predict the survival time of patients with stage III colorectal cancer. Shibutani et al. (54) examined the infiltration of tumor infiltrating lymphocytes (TILs) in the primary tumors of patients with stage IV CRC, according to the method proposed by the international TILs working group. They found that patients with high TILs had a higher response rate to chemotherapy than patients with low TILs (79.3% vs. 48.1%, respectively, *P* < 0.025). Furthermore, patients with high TILs had higher OS times than those with low TILs (median survival time 35.5 months and 22.4 months respectively, *P* < 0.0221). In addition, Emile et al. (55) discussed the prognostic value of TILs in 1,220 patients with stage III CRC who received folinic acid, fluorouracil, and oxaliplatin (FOLFOX) chemotherapy. They found that the recurrence rate of the high TILs group was 14.4%, whereas the recurrence rate of the low TILs group was 21.1% (*P* = 0.020). Patients with high TILs had higher OS and disease-free survival (DFS) than patients with low TILs. Thus, the level of lymphocyte infiltration into the tumor microenvironment may affect the efficacy treatment regimens and can predict the prognosis of CRC patients to an extent. M1 macrophages secrete tumor necrosis factor-α (TNF-α), which can kill and inhibit the growth of tumor cells. M1 macrophages highly express major histocompatibility complex (MHC) class I and class II molecules, which present tumor-specific antigens and indirectly inhibit the growth of tumor cells (52). Therefore, differences in M1 macrophage levels could explain why patients with high-ADI CRC have significantly shortened OS times. In addition, we found that the activity of some carcinogenesis pathways, such as fibroblast growth factor signaling, angiogenesis, ROS synthesis processes, JAK/STAT signaling, and IL-1 production, was significantly upregulated in the high-ADI group compared to the low-ADI group (56–58). The activated MAPK and PI3-K pathways have been shown to regulate gene expression and protein expression, thus promoting cell growth and proliferation and reducing apoptosis (56). IL-1 and IL-6 have been shown to have multiple effects on multiple cell types, and these cytokines participate in various stages of tumor formation, invasion and metastasis. IL-1 and IL-6 can regulate the carcinogenic transcription factors NF-κB and STAT3, which play important biological functions and can accelerate tumor growth and progression (59). In addition, IL-6 can promote platelet production (60), and activated platelets can act as chemotactic agents for tumor cells, promoting the formation of metastatic lesions and increasing the level of circulating tumor cells (57, 58). High levels of VEGF can promote the growth, angiogenesis, and metastasis of tumor cells (61–63). A chronic inflammatory microenvironment can promote cell proliferation and cause uncontrolled growth, which leads the formation of tumors. Inflammation can also promote tumor progression through genomic instability, initial infiltration, and metastasis (64). In addition, studies have shown that fibroblast growth factor-2 (FGF-2) regulates tumor angiogenesis (65). Tumors often originate from sites of chronic inflammation or infection, and large numbers of phagocytes can induce oxidative stress by producing ROS and reactive nitrogen species (RNS) after infiltrating the site of inflammation, thus causing DNA damage in the host tissues. Therefore, persistent inflammation can promote the accumulation of mutations (66–68). For this reason, significant upregulation of carcinogenesis-associated pathways in high-ADI patients could explain their significantly shortened OS times.

In the present study, we found that high-ADI may worsen the prognosis of CRC, and there was no correlation between BMI and prognosis of TCGA-CRC (Figure 2D; log-rank *P* > 0.05). In the correlation analysis, we found that there was no significant relationship between ADI and BMI (Figure 2C, *P* > 0.05). At present, there is controversy about the relationship between BMI and patient prognosis (69). Many studies have shown that there is no significant relationship between BMI and prognosis (70–72); however, some studies have shown that BMI is positively correlated with mortality (4, 44). Therefore, we suspect that BMI may have a different effect than adipose tissue on the occurrence and progression of CRC. Adipose tissue is an important endocrine organ that can induce and secrete a variety of endocrine factors and fat factors, thus exerting anti-inflammatory and pro-inflammatory functions (73). Moreover, obese patients often have increased leptin, decreased adiponectin, and increased pro-inflammatory adipokines (74). Leptin activates the JAK/STAT pathway by binding its receptor (OB-R) and thereby promotes the adhesion and invasion of colorectal cancer cells (75). On the other hand, adiponectin can inhibit the proliferation of cancer cells and induce apoptosis (76).

In the present study, ADI obtained from deep learning analysis of CRC patient H&E images could be used as a potential biomarker for the clinical prognosis of a CRC patient. However, there were limitations in the present study. First, when training the model, many samples needed to be included, and the samples needed to be cut into many small squares; therefore, building the model required considerable computer space and operation time. However, when the completed model was used to interpret a patient's H&E slice, only a few seconds were needed to obtain classification results. Second, large images require large amounts of memory, and consequently, calculations are complicated and slow. For this reason, we segmented images into smaller pictures. When segmenting images, we tried our best to ensure accuracy, but some errors may still have occurred. Accuracy will therefore be improved if a pathologist performs secondary screening based on the small cut pictures. Third, the ADI constructed by the CNN model is only aimed at predicting OS, but not other prognosis metrics, such as progression-free survival (PFS), disease-free survival (DFS), and DSS. Fourth, we could not assess the association between ADI and known prognostic factors, such as tumor budding, number of lymph nodes, tumor location, microsatellite instability, TILs, mutations (such as BRAF and KRAS), or histological subtypes. Fifth, in the two independent verification sets, the predictive value of ADI for subgroup prognosis was not entirely consistent. Therefore, future studies should include a larger sample of queues for verification.

## Conclusions

In the present study, we used a CNN model to automatically quantify ADI for H&E-stained CRC images. Furthermore, we found that ADI may predict OS in CRC patients and their subgroups (COAD, READ, CRC-Female and CRC-Male). The present study shows that automatic histopathological image analysis can be achieved through a deep learning model. The quantified image features could assist with predicting patient prognosis and guide clinical decision-making.

## Data Availability Statement

The original contributions presented in the study are included in the article/Supplementary Material, further inquiries can be directed to the corresponding authors.

## Ethics Statement

The studies involving human participants were reviewed and approved by Zhujiang Hospital of Southern Medical University and First People's Hospital of Chenzhou. The patients/participants provided their written informed consent to participate in this study. Written informed consent was obtained from the individual(s) for the publication of any potentially identifiable images or data included in this article.

## Author Contributions

PL and JZ: conceptualization. AL, CQ, and ML: formal analysis and visualization. AL, CQ, ML, RG, QC, ZL, XW, QL, JZ, and PL: writing—original draft. AL, CQ, ML, RG, EI, NM, QC, ZL, XW, QL, JZ, and PL: writing—review and editing. All authors read and approved the final manuscript.

## Funding

This work was supported by the Natural Science Foundation of Guangdong Province (Grant Nos. 2018A030313846 and 2021A1515012593), the Science and Technology Planning Project of Guangdong Province (Grant No. 2019A030317020), and the National Natural Science Foundation of China (Grant Nos. 81802257, 81871859, 81772457, 82172750, and 82172811).

## Conflict of Interest

The authors declare that the research was conducted in the absence of any commercial or financial relationships that could be construed as a potential conflict of interest.

## Publisher's Note

All claims expressed in this article are solely those of the authors and do not necessarily represent those of their affiliated organizations, or those of the publisher, the editors and the reviewers. Any product that may be evaluated in this article, or claim that may be made by its manufacturer, is not guaranteed or endorsed by the publisher.

## Supplementary Material

Supplementary Table 1 Detailed clinical information for TCGA-CRC.

Supplementary Table 2 Detailed clinical information for Local-CRC1.

Supplementary Table 3 Detailed clinical information for Local-CRC2.
