# Supplementary figures and images for: Deep Learning Analysis of the Adipose Tissue and the Prediction of Prognosis in Colorectal Cancer
Source: Front Nutr. 2022 May 11;9:869263. doi: 10.3389/fnut.2022.869263 (PMC9131178; doi:10.3389/fnut.2022.869263)

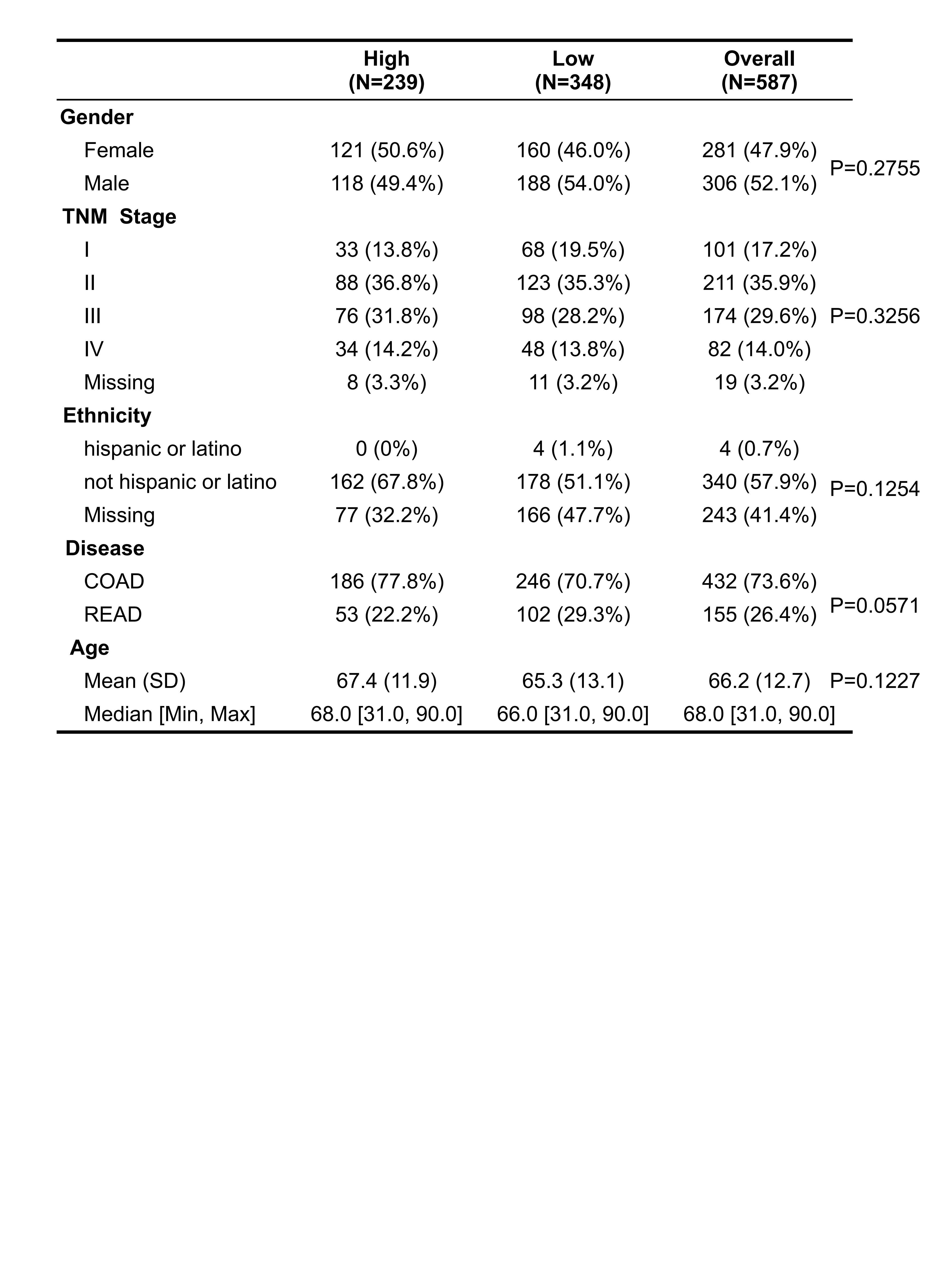

Supplement: Supplementary Table 1 — Detailed clinical information for TCGA-CRC. [file Image_1.JPEG]

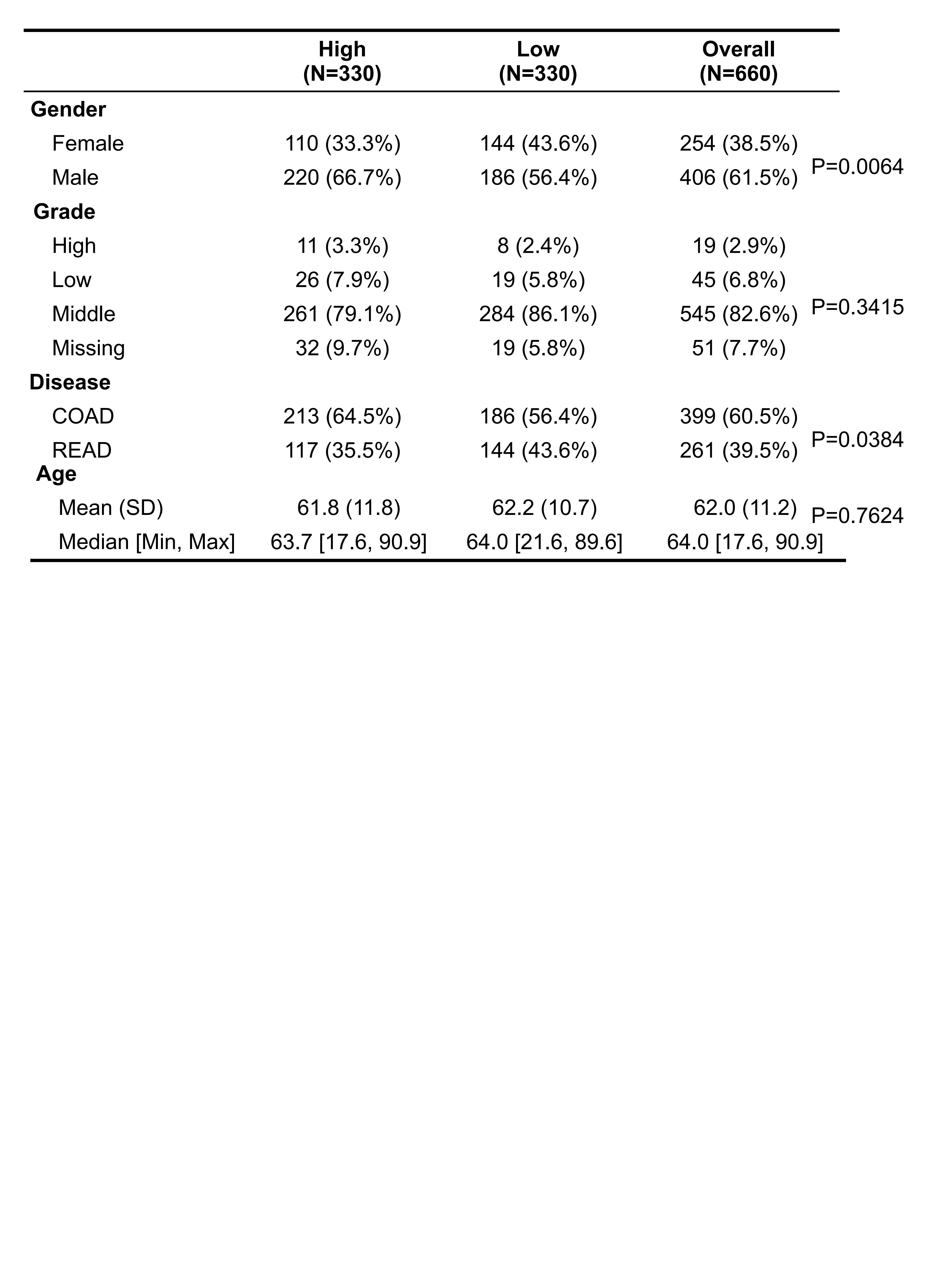

Supplement: Supplementary Table 2 — Detailed clinical information for Local-CRC1. [file Image_2.JPEG]

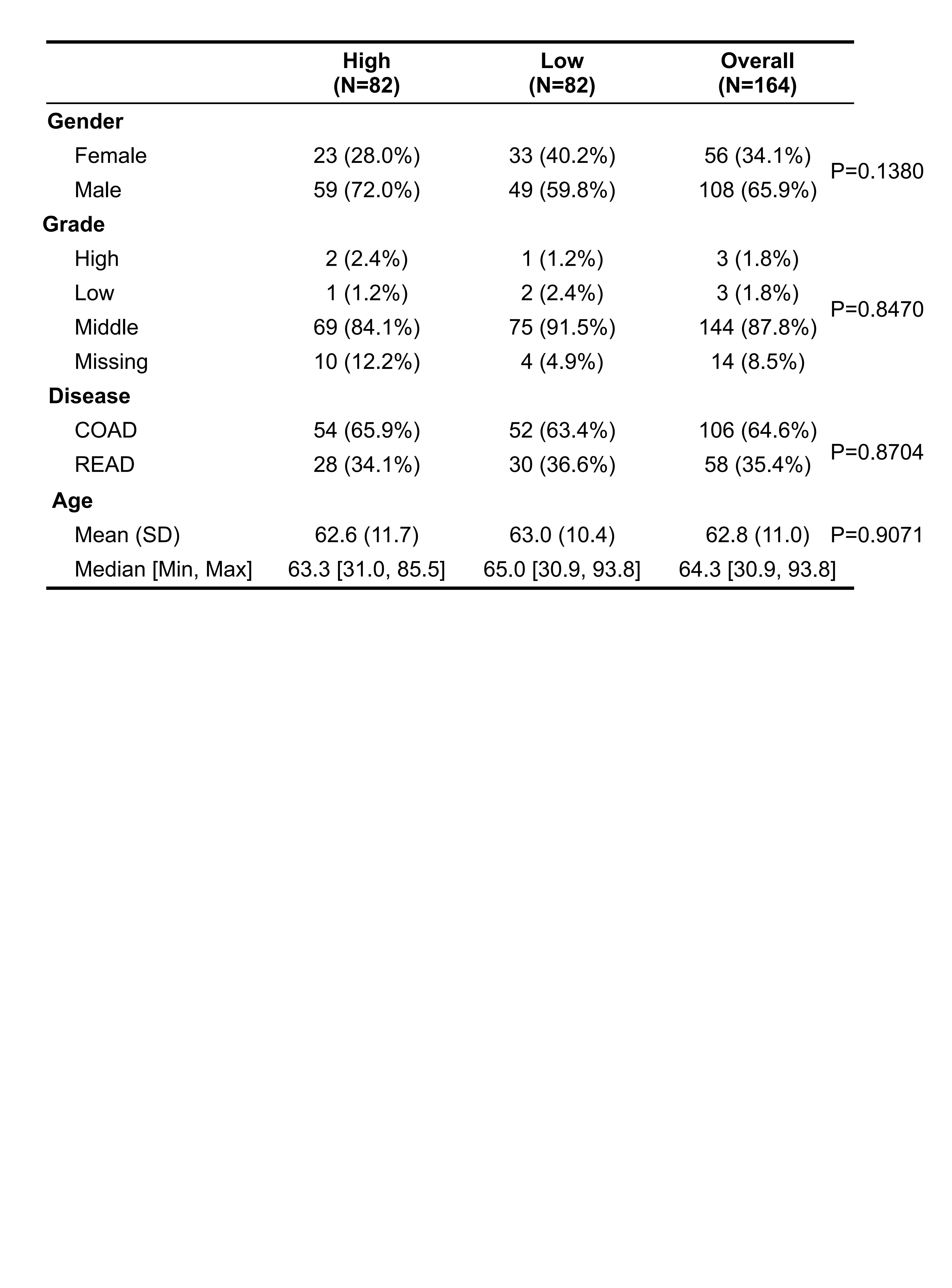

Supplement: Supplementary Table 3 — Detailed clinical information for Local-CRC2. [file Image_3.JPEG]
